# Natural Polypropionates in 1999–2020: An Overview of Chemical and Biological Diversity

**DOI:** 10.3390/md18110569

**Published:** 2020-11-19

**Authors:** Zhaoming Liu, Hongxin Liu, Weimin Zhang

**Affiliations:** State Key Laboratory of Applied Microbiology Southern China, Guangdong Provincial Key Laboratory of Microbial Culture Collection and Application, Guangdong Open Laboratory of Applied Microbiology, Guangdong Institute of Microbiology, Guangdong Academy of Sciences, 100 Central Xianlie Road, Yuexiu District, Guangzhou 510070, China; liuzm@gdim.cn (Z.L.); liuhx@gdim.cn (H.L.)

**Keywords:** polypropionate, marine organism, chemical diversity, bioactive diversity, biodiversity

## Abstract

Natural polypropionates (PPs) are a large subgroup of polyketides with diverse structural features and bioactivities. Most of the PPs are discovered from marine organisms including mollusks, fungi and actinomycetes, while some of them are also isolated from terrestrial resources. An increasing number of studies about PPs have been carried out in the past two decades and an updated review is needed. In this current review, we summarize the chemical structures and biological activities of 164 natural PPs reported in 67 research papers from 1999 to 2020. The isolation, structural features and bioactivities of these PPs are discussed in detail. The chemical diversity, bioactive diversity, biodiversity and the relationship between chemical classes and the bioactivities are also concluded.

## 1. Introduction

Natural polypropionates (PPs) are a large subgroup of polyketides constructed by C_3_-units. Most of the PPs are discovered from marine organisms including mollusks, fungi and actinomycetes, while some of them are also isolated from terrestrial resources. One of the main characteristics to distinguish the PPs is the regularly interspaced methyl groups in the polyketide chain or the cyclic polyketide core, which are driven directly from propionate unit or from the acetate-methionine motif. Due to their flexible biosynthetic connections, polypropionate derivatives always perform abundant structural diversities. Moreover, they also play as important building blocks in the biosynthesis of several kinks of antibiotics such as macrolides, polyether and cyclic peptides. While some of the polypropionate metabolites exert an ecological influence in the organisms, most of them have been demonstrated to exhibit various kinds of bioactivities, especially antitumor and antimicrobial effects [1]. The first propionate-derived metabolite was isolated from marine mollusk *Tridachiella diomedea* in 1978, which was identified as tridachiosne through X-ray diffraction by C. Ireland and D.J. Faulkner. [2] Since then, there has been an increasing number of PPs with different structural features reported from nature or by genomic engineering. However, the only review in the past reported by Michael T. Davies-Coleman and Mary J. Garson summarized 168 PPs from marine resources up to the end of 1997 [3] and there is no additional updated review in the past two decades.

**Figure 1:**
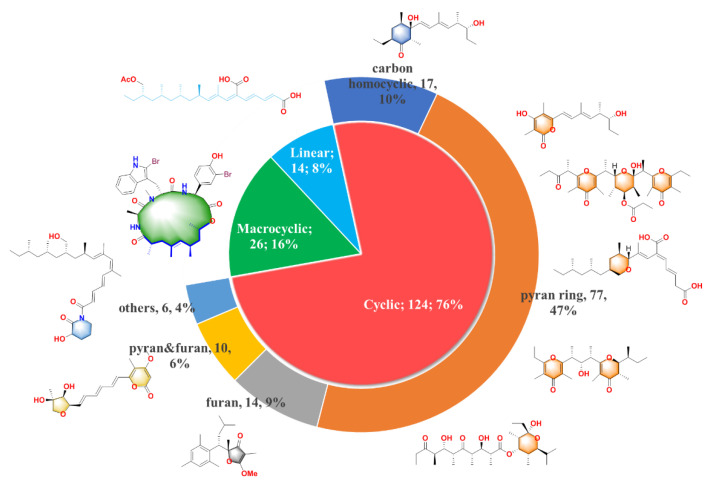
164 natural polypropionates were divided into three main groups; the cyclic polypropionates (PPs) were further classified into five sub-groups; the characteristic chemical classes are highlighted in different colors.

In the current review, we focus on the chemical structures and bioactive properties of the new PPs discovered from both marine and terrestrial systems in 1999–2020. A total of 67 research papers containing 164 new PPs (74% of which were from marine resources), which are driven from either the putative polypropionate-related biosynthesis pathway or the mixed biogenetic pathway involving propionate units, have been summarized in this review. The new compounds can be divided into three main groups according to their structure features: the first one is linear molecular (8%), the second one is cyclic polypropionate (76%) and the last one is macrocyclic derivatives (16%). Moreover, it can be noticed that the cycle containing PPs is the most abundant group with the largest proportion, which can be further distributed into five subcategories shown in the pie chart of Figure 1: carbocyclic (10%), pyran derivatives (47%), furan derivatives (9%), pyran and furan containing (6%) and other cyclic analogues (4%). We will present their isolation, structure and bioactivities in detail; meanwhile, the chemical diversity, bioactive diversity and the biodiversity are also discussed in this review.

## 2. Isolation, Structural Features and Bioactivities of Polypropionates

### 2.1. Linear Metabolites

**Figure 2:**
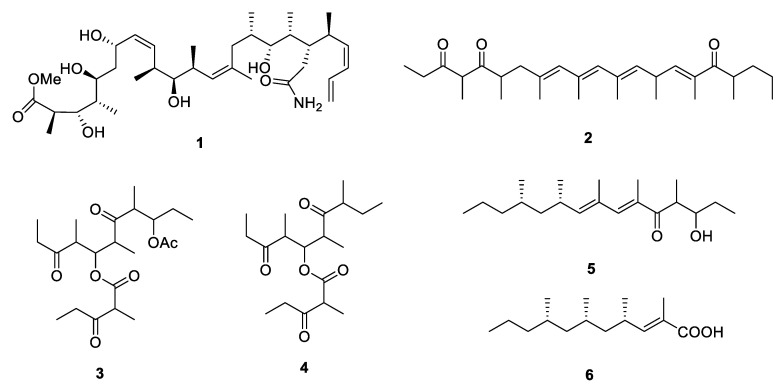
Chemical structures of **1**–**6**.

**Figure 3:**
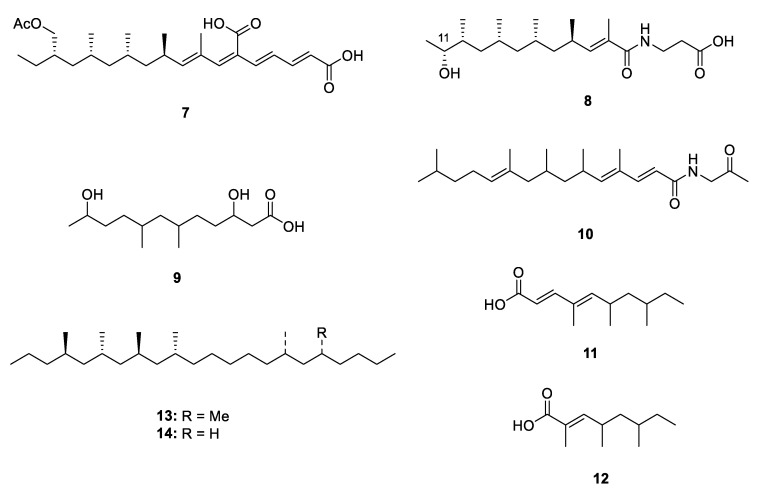
Chemical structures of **7**–**14**.

The linear polypropionate derivatives are a relatively rare group discovered from nature, which are driven directly from propionate pathway or acetate-methionine pathway. From 1999 to 2020, 14 linear analogues have been isolated from marine mollusks, microorganisms, terrestrial plants and insects (Figure 2 and Figure 3).

Caribbean marine sponge *Discodermia dissolute* collected from Grand Bahama Island yielded an amide containing polypropionate 5-hydroxymethyldiscodermolate (**1**) [4] with 13 chiral centers. The absolute configuration was determined by a comparison of the NMR data with those of the methanolysis product derived from the known polypropionate discodermolide [5]. Compound **1** exhibited strong cytotoxicity against murine P388 leukemia and human lung adenocarcinoma A549 cell lines with the IC_50_ values of 65.8 and 74 nM, respectively. Exiguaone (**2**) [6] was isolated from the lipidic extract of Mediterranean cephalaspidean mollusk *Haminoea exigua* while micromelones A and B (**3** and **4**) [7] were isolated from marine gastropod *Micromelo undata*. The absolute configuration of them remains unknown. The chemical investigation of two species of South African mollusk *Siphonaria* led to the discovery of two new linear PPs, (6*E*,8*E*,10*S*,12*S)*-3-hydroxy-4,6,8,10,12-pentamethylpentadeca-6,8-dien-5-one (**5**) and (2*E*,4*S*,6*S*,8*S*)-2,4,6,8-tetramethyl-2-undecenoic acid (**6**) [8,9]. The stereochemistry of **6** was further elucidated by oxidative degradation.

Microorganisms, especially the terrestrial fungi, are the potential source of natural PPs. Six new PPs, including two linear ones, fiscpropionates C and F (**7** and **8**) [10], were isolated from the deep-sea-derived fungus *Aspergillus fischeri*, which represented the first discovery of polypropionate derivatives from the deep-sea-derived fungus. The absolute configuration at C-11 of **8** was deduced by the modified Mosher’s method. In the bioassays, **7** was detected to show strong noncompetitive inhibitory effects against *Mycobacterium tuberculosis* protein tyrosine phosphatase B (*M*ptpB) with the IC_50_ value of 4.0 µM. A limestone soil-derived fungus *Penicillium decumbens* yielded an aliphatic acid 3,11-dihydroxy-6,8-dimethyldodecanoic acid (**9**) [11] which constructed a polypropionate fragment in the aliphatic chain. The bioassay-guided fractionation of the actinobacterium *Saccharothrix xinjiangensis* collected from Caspian Sea beach led to isolation of a N-containing polypropionate saccharonoic acid (**10**), which exhibited weak inhibitory activity against *Mucor hiemalis* and *Candida albicans* (IC_50_: 66.7 and 33.4 µg/mL) [12]. Xylarinic acids A and B (**11** and **12**) were antifungal PPs from the fruiting body of *Xylaria polymorpha* [13].

Two linear metabolites were also isolated from a terrestrial insect. 4,6,8,10,16,18-hexa- and 4,6,8,10,16-pentamethyldocosanes (**13** and **14**) were two major methylated hydrocarbons obtained from cane beetle species *Antitrogus parvulus* [14,15], and their absolute configuration was established by a series of chemical conversion, chromatographic and spectroscopic comparisons.

### 2.2. Cyclic Metabolites

#### 2.2.1. Carbon Homocyclic Metabolites

PPs containing a carbon homocycle are always driven from the mixed biogenetic pathway. They usually present a five-/six-member ring or the benzene ring. The complicated ring systems such as spirocyclic or bridged cyclic are also discovered but quite rare in polypropionate derivatives.

**Figure 4:**
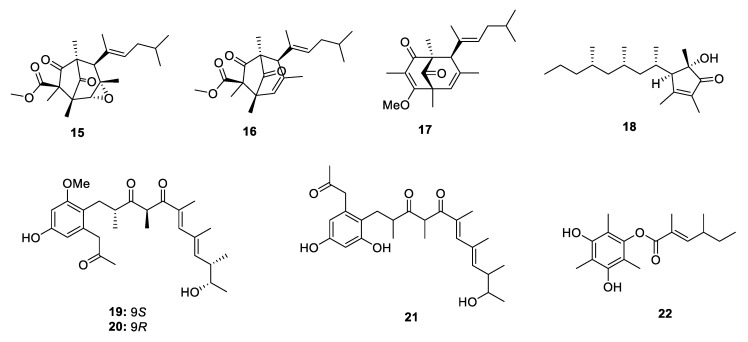
Chemical structures of **15**–**22**.

Three pairs of enantiomers (±)-ocellatusones A–C (**15**–**17**), constructing a bicyclo[3.2.1]octane or bicyclo[3.3.1]nonane, were discovered from a South-China-Sea-derived photosynthetic mollusk *Placobranchus ocellatus* [16]. These represent the most complex carbon homocycle containing PPs to date (Figure 4). The optically pure enantiomers of the racemic compounds were obtained by chiral HPLC resolution. Furthermore, the biomimetic semisynthesis of **15** from a known polypropionate tridachiahydropyrone [17] was performed through ZnCl_2_ catalysis, which confirmed the new and diversity-generating rearrangements from the same precursor in the biosynthetic pathway. A mollusk *Siphonaria capensis* collected from the intertidal zone at the Bushman’s River mouth yielded a cyclopentanone-derived polypropionate capensinone (**18**) (Figure 4) [9].

**Figure 5:**
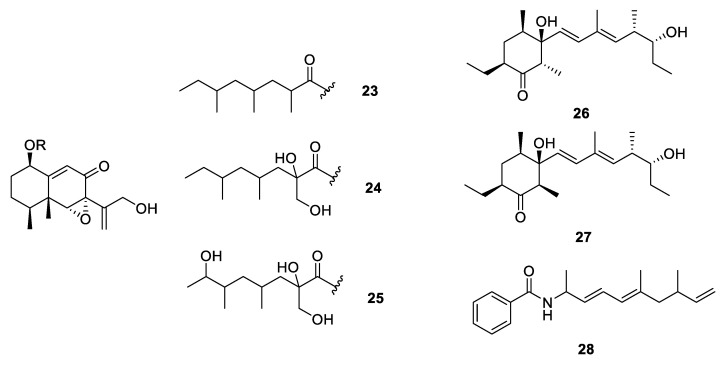
Chemical structures of **23**–**28**.

Ten carbon homocyclic PPs were obtained from microorganisms including fungi, actinomycetes and myxobacteria (Figure 4). Porosuphenols A–C (**19**–**21**) were isolated from a marine-derived *Aspergillus porosus* [18], and asperolan (**22**) was isolated from a *Garcinia preussii* endophytic fungus *Aspergillus japonicus* [19]. The absolute configuration of **19** was established using NMR data guided conformer searching and electrostatic circular dichroism (ECD) calculations. A *Camarops*-like endophytic fungus collected from *Alibertia macrophylla* yielded three eremophilane sesquiterpene derivatives xylarenones C–E (**23**–**25**) which constructed a polypropionate side chain (Figure 5) [20]. In the bioactivity tests, only **23** displayed strong enzyme inhibition activities against subtilisin and pepsin protease with the IC_50_ values of 0.462 and 0.288 µM, respectively. Further, pacificanones A and B (**26** and **27**) were produced by a marine actinomycete *Salinispora pacifica* (Figure 5) [21], while a new cytotoxic polyketide synthase–nonribosomal peptide synthetase (PKS-NRPS) hybrid polypropionate, haliamide (**28**) was isolated from a marine myxobacterium *Haliangium ochraceum* and showed cytotoxicity against HeLa-S3 cells (IC_50_ = 12 µM) [22].

#### 2.2.2. Pyran-Related Metabolites

**Figure 6:**
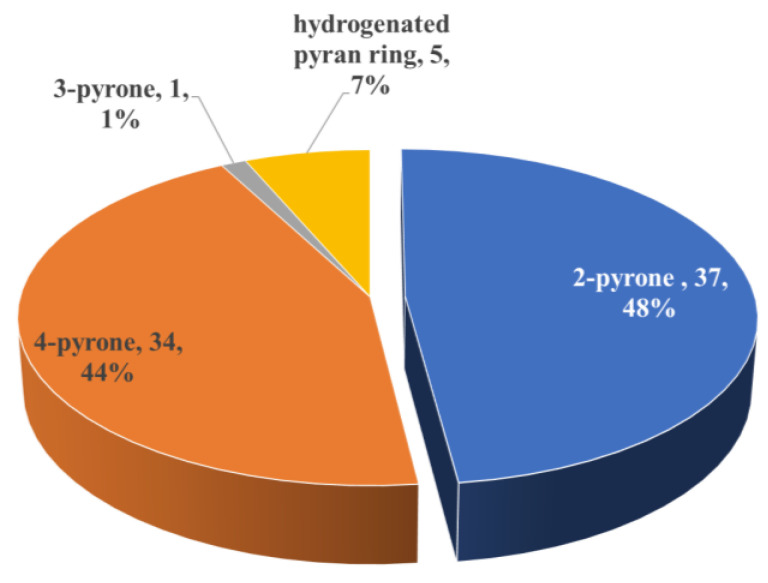
Classification of pyran-related PPs.

**Figure 7:**
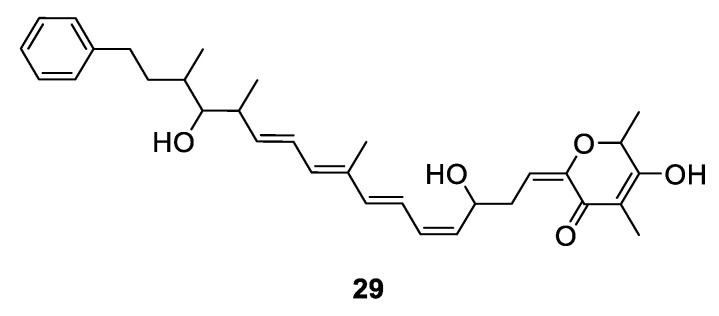
Chemical structures of **29**.

Pyran ring is very common in PPs and nearly half (47%) of the naturally occurring PPs possessing one or more pyran or pyran-related cycle(s). A total of 77 new PPs were concluded in this section (Figure 6), including 37 metabolites containing a 2-pyrone ring, 34 metabolites containing 4-pyrone ring(s), 5 metabolites containing a hydrogenated pyran ring and a derivative containing an unusual 3-pyrone moiety (Figure 7, hyapyrones A **29** from myxobacteria *H. minutum* [23]).

##### Metabolites Containing 2-Pyrone(s)

**Figure 8:**
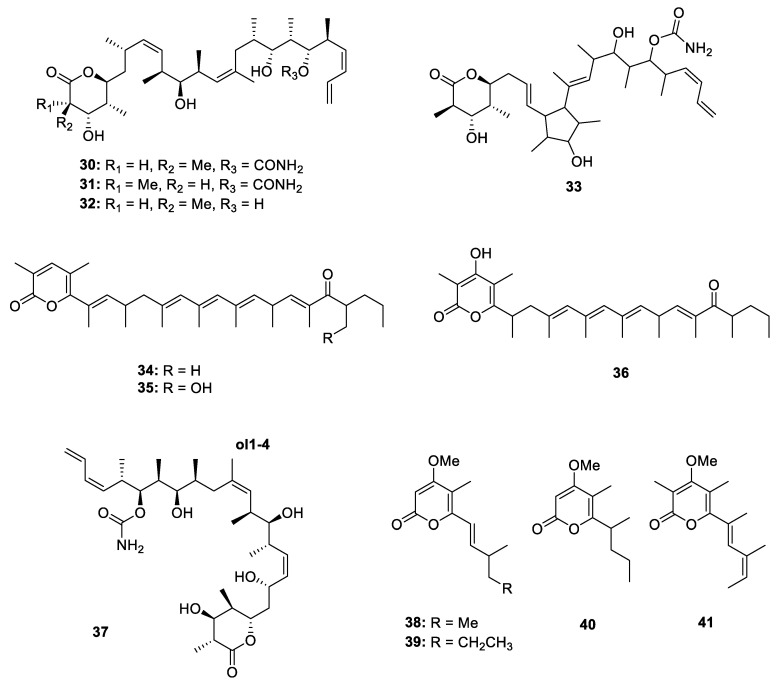
Chemical structures of **30**–**41**.

The marine sponge *Discodermia* sp. has yielded a linear polypropionate (**1**) together with four 2-pyrone-containing series **30**–**33** (Figure 8). All of them showed strong in vitro inhibitory activity against proliferation of the P-388 cell line with the IC_50_ values in the range of 0.13–5.0 µM [4]. Fusaripyrones A and B (**34** and **35**) were isolated from Mediterranean mollusk *Haminoea fusari* [24] while exiguapyrone (**36**) was isolated from mollusk *H. exigua* [6]. (+)-Discodermolide (**37**) was obtained from a sponge *D. dissolute* previously and its diverse pharmacological activities made it to be a synthetic target for chemists. The solution structure of **37** was determined via NMR techniques and conformational analysis by Amos B. Smith III and his co-workers for the first time, which demonstrated that **37** occupies a helical conformation in solvent [25,26,27,28,29,30]. Four α-pyrones propionates (**38**–**41**) were produced by mediterranean sacogolssan *Placida dendritica* (Figure 8).

**Figure 9:**
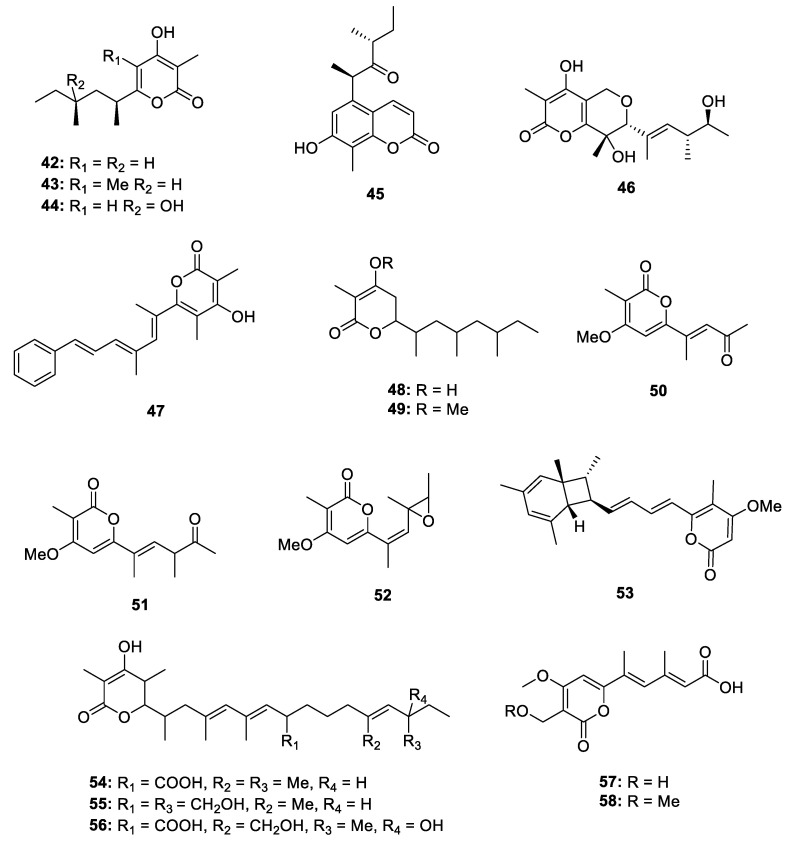
Chemical structures of **42**–**58**.

**Figure 10:**
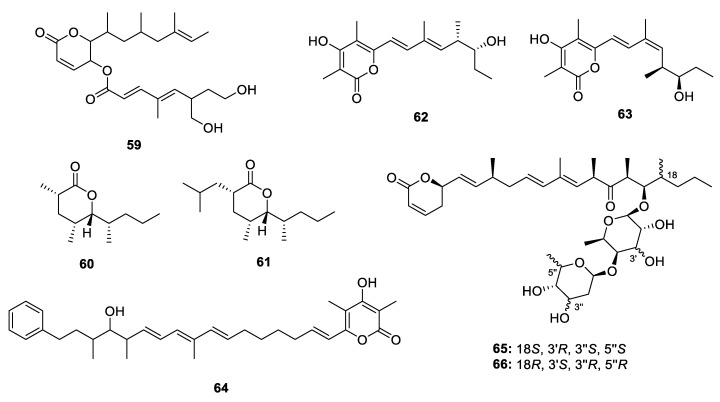
Chemical structures of **59**–**66**.

The fungi are the main producer of 2-pyrone-containing PPs (Figure 9). Three different species of *Aspergillus* genus yielded five metabolites, including nipyrones A‒C (**42**–**44**) [31], versicolone A (**45**) [32] and neovasinin (**46**) [19]. The stereochemistry of the chiral centers at the side chain of **42**–**44** was deduced by the NOE correlations and ECD calculations while the 9*R*, 11*R* configuration of **45** was established by comparing the optical rotation between natural isolates and ascosalitoxin isomers. The absolute configuration of **45** was directly confirmed by X-ray crystallographic analysis. In the bioactivity tests, **44** not only showed promising activity against *Staphylococcus aureus* and *Bacillus subtilis* with the MIC values of 8 and 16 µg/mL, respectively, but also displayed weak antitubercular activity against *M. tuberculosis*, with an MIC value of 64 µg/mL. Bioassay-guided investigation of another plant-associated fungus *Aspergillus* sp. led to the isolation of a new enzyme inhibitor aspopyrone A (**47**) [33], which exhibited significant Protein Tyrosine Phosphatase 1B (PTP1B) and T-cell PTP inhibitory activities with the IC_50_ values of 6.7 and 6.0 µM. Two new α-pyrones fupyrones A and B (**48** and **49**) were isolated from an endophytic fungus *Fusarium* sp. [34] while three 2-pyrone phomapyrones D‒G (**50**–**52**) were identified from the mixed extract of pathogen *Leptosphaeria maculans*. Using the incorporations of ^13^C-labeled acetate/malonate and deuterated methionine led to the illustration of an acetate-methionine biosynthetic pathway of phomapyrones [35]. An endophytic *Penicillium* sp. from *Catharanthus roseus* yielded a novel bicyclo[4.2.0]octadiene containing polypropionate citreoviripyrone A (**53**) [36]. Interestingly, using the Zn(II)-type and NAD^+^-dependent histone deacetylase inhibitors in fermentation could significantly enhance the production of **53** in this strain. A polypropionate-related *alp* gene cluster was shown to be almost silent in wheat pathogen *Parastagonospora nodorum*, but it could be significantly upregulated when reconstructed heterologously in *Aspergillus nidulans*. Based on the point, three new 2-pyrone PPs alternapyrones B‒D (**54**–**56**) were obtained [37]. The bioactivity screening indicated that **54** displayed potential antibacterial activity against *B. subtilis*, anti-parasitic activity against *Giadia duodenalis* and cytotoxicity against murine myeloma. Chemical investigation of the other two unusual fungi *Stemphylium* sp. and *Talaromyces* sp. led to the isolation of infectopyrones A and B (**57** and **58**) [38] as well as rasfonin (**59**) (Figure 10) [39]. **57** and **58** had a broad spectrum of antibacterial activity against five terrestrial pathogenic bacteria while rasfonin could induce cell death in Ba/F3-V12 cells with the IC_50_ of 0.16 µg/mL.

Seven PPs were isolated from several antinomycetes, myxobacteria and slime molds (Figure 10), including **60** and **61** from marine-sediment-derived *Nocardiopsis tangguensis* [40], salinipyrones A and B (**62** and **63**) from marine-derived *Salinispora pacifica* [21], hyapyrones B (**64**) from the myxobacterium *H. minutum* [23] and lycogalinosides A‒B (**65**–**66**) from slime mold *Lycogala epidendrum* [41]. The absolute configuration of **60** and **61** was established by NOESY analysis followed by hydrolysis and Mosher’s method. Further, **64**, **65** and **66** exhibited antibacterial activities against Gram-positive bacteria.

##### Metabolites Containing 4-Pyrone(s)

**Figure 11:**
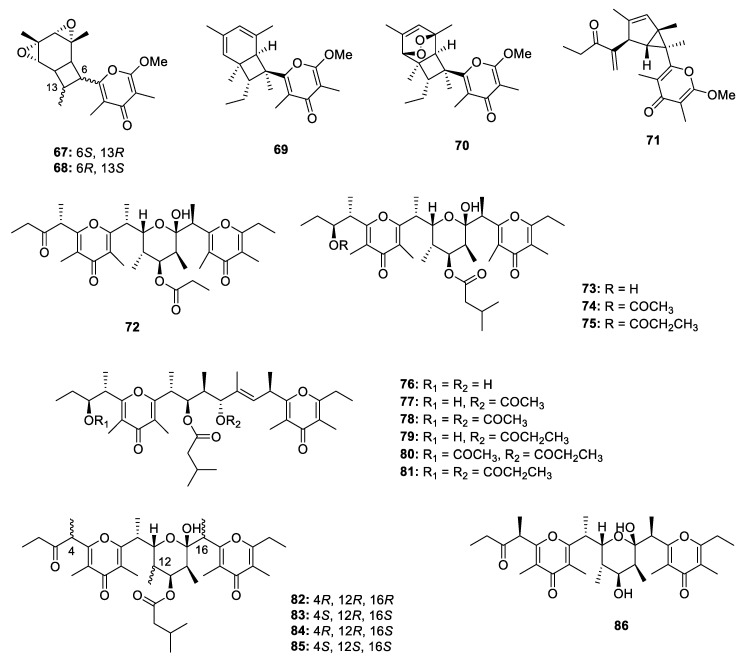
Chemical structures of **67**–**86**.

**Figure 12:**
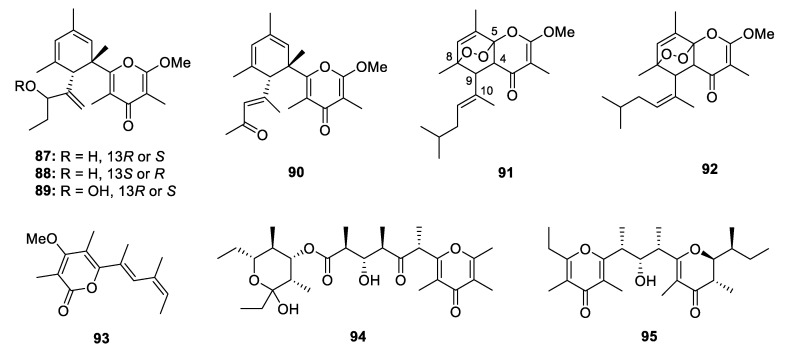
Chemical structures of **87**–**95**.

**Figure 13:**
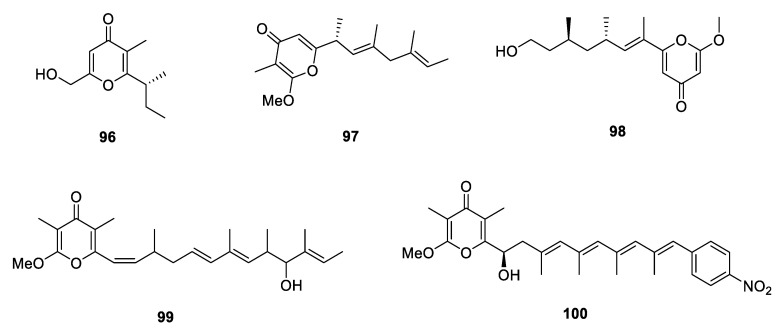
Chemical structures of **96**–**100**.

A total of 34 4-pyrone-containing PPs were discovered (Figure 11, Figure 12 and Figure 13), 29 of which were produced by mollusks. The structural features of 4-pyrone PPs are more diverse than those of 2-pyrone derivatives. In general, 2-pyrone PPs construct an α-pyrone core and a propionate chain, while the 4-pyrone PPs always form two or three 4-pyrone moieties through dehydration.

The study of Pacific sacoglossan *Elysia diomedea* led to isolation of two propionates elysiapyrones A and B (**67** and **68**), constructing a bicyclo[4.2.0]octane core and a substituted 4-pyrone moiety. It was speculated that the bicyclo[4.2.0]octane core was formed through an enzymatic intramolecular [2+2]-cycloadditions [42]. A similar analogue (**69**) without the epoxide moieties was isolated from another species of sacoglossan *Placobranchus ocellatus* together with its peroxy derivative (**70**) [43]. In the same year, Aubry K. Miller and Dirk Trauner completed the total synthesis of **69** and **70** [44]. The sacoglossan *Elysia patagonica* yielded a main constituent phototridachiapyrone J (**71**) [45], which is an isomer of bicyclo[4.2.0]octane propionate by constructing an bicyclo[3.1.0]octane core. Fifteen muti-pyrones containing PPs **72**–**86**, belonging to onchidione family, were isolated from four species of *Onchidium* [46,47,48,49,50,51]. **76**–**81** possess a propionate chain with 4-pyrones at both sides. In the antitumor assays, the isolated onchidione derivatives showed moderate to strong inhibitory effects against different kinds of human cancer cell lines, and compound **83** was further detected to show significant activation on the splicing of the XBP1 mRNA by 217.8% at 10 µg/mL. Chemical investigation of a sacoglossan mollusk *Placobranchus ocellatus* collected in the Philippines led to the isolation of four new tridachione-type metabolites tridachiapyrones G‒J (**87**–**90**) as well as a mixture of peroxide containing tridachiahydropyrones B‒C (**91** and **92**) [52]. For compounds **87**–**90**, only the relative configuration was determined by NOESY analysis. **91** and **92** were isomers at the double bond Δ^10^ but the stereochemistry at C-4, C-5, C-8 and C-9 has not been identified. The peroxide group was confirmed by EIMS fragmentation analysis. Another peroxide containing polypropionate **93** was isolated from mantle extract of *Placida. dendritica* [53]. An unusual non-contiguous polypropionate siserrone A (**94**) was obtained from *Siphonaria serrata* and its relative configuration was determined by a combination of ROESY and H-H coupling constant analysis. The absolute configuration could not be identified because **94** was unstable and easy to be degraded [54]. The Caribbean sponge *Smenospongia aurea* yielded a bis-γ-pyrone polypropionate smenopyrone (**95**) [55]. The stereochemistry of the pyrone ring was identified by comparing the *J* values with those of the known compounds containing similar moiety, while the configuration at C-9 and C-10 was determined through ^13^C NMR calculations. A further ECD analysis led to the establishment of the absolute configuration of **95**.

Three new simple 4-pyrone PPs, **96**–**98**, were isolated from *Aspergillus versicolor*, *Acremonium citrinum* and *Fusarium solani*, respectively [32,56,57]. **98** displayed a dose-dependent neuroprotective effect against glutamate-induced cytotoxicity in HT22 murine hippocampal neuronal cells. Besides, two 4-pyrone propionates, **99** and **100**, constructed a relatively long side chain, were produced by an actinomycete *Streptomyces* sp. [58,59]. **99** dose-dependently inhibited luciferase expression in 2-deoxyglucose treated HT1080 human fibrosarcoma cells (IC_50_ = 7.8 nM) and it could also inhibit GRP78 protein expression and induce cell death under endoplasmic reticulum stress. **100** exhibited moderate cytotoxicity against HCT-116, HepG2 and A549 cell lines with the IC_50_ values in the range of 3.0–6.0 µg/mL.

##### Metabolites Containing Hydrogenated Pyran(s)

**Figure 14:**
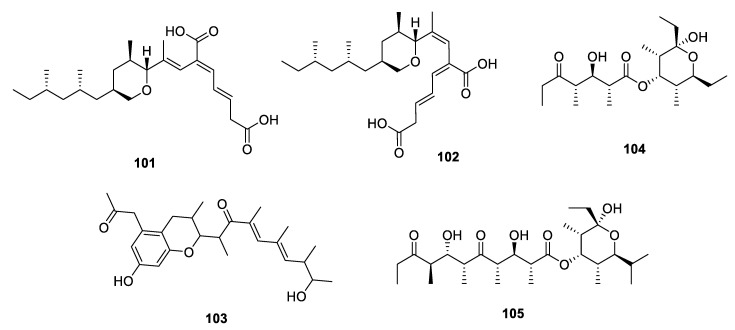
Chemical structures of **101**–**105**.

The deep-sea-derived fungus *A. fischeri* yielded two tetrahydropyran-derived PPs fiscpropionates A and B (**101** and **102**) (Figure 14). The stereochemistry of side chain was deduced by the *J*-HMBC experiments, which was the first example to use the C-H coupling constants to solve the configuration of PPs. Both **101** and **102** exhibited significant *M*ptpB inhibition activities through a noncompetitive mechanism with the IC_50_ values of 5.1 and 12 µM, respectively. The quantitative structure-activity relationship (QSAR) analysis suggested that the terminal hydrophilic functional group as well as an opposing hydrophobic chain would play an important role in *M*ptpB inhibition activities [10]. Another marine-derived fungus *A. porosus* produced three new PPs porosuphenols A‒C (**19**–**21**) and a hydrogenated benzopyran derivative porosuphenol D **103** (Figure 14) [18]. Though a series of conformation analysis was adapted to solve the stereochemistry of **19** and **20**, the attempts to assign the absolute configuration of **21** and **103** were unsuccessful. Dolabriferols B and C (**104** and **105**) were two propionate-related metabolites from Caribbean mollusk *Dolabrifera dolabrifera* collected from Puerto Rico (Figure 14) [60]. The absolute configuration was established by a combination of X-ray diffraction and chemical degradation studies.

#### 2.2.3. Furan-Related Metabolites

**Figure 15:**
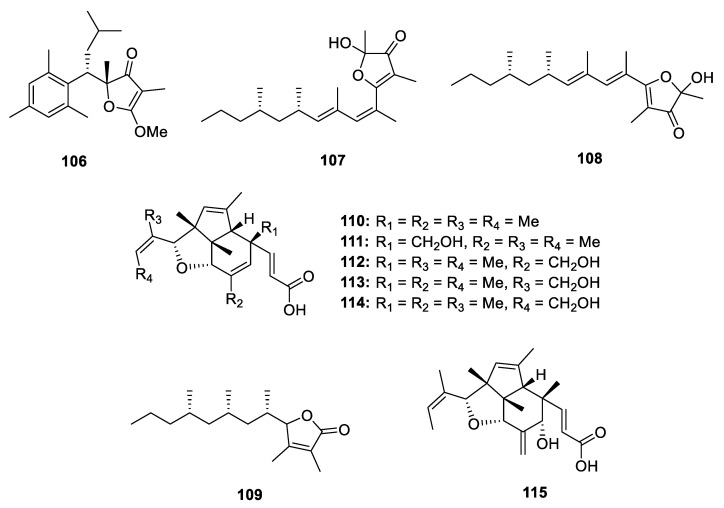
Chemical structures of **106**–**115**.

Except for the three pairs of carbon homocyclic PPs (±)-**15**–(±)-**17**, the mollusk *P. ocellatus* also produced a pair of furanone-containing analogues (±)-**106** [16], which represents a unique dimethylfuran-3(*2H*)-one nucleus connected with a mesitylene moiety (Figure 15). An X-ray diffraction was carried out to confirm its racemic nature by a *P-1* space group. 6*Z*,8*E*-Δ^8^-Siphonarienfuranone (**107**) and 6*E*,8*E*-Δ^8^-siphonarienfuranone (**108**) are two new epimers at Δ^6^ produced by the mollusk *Siphonaria oculus* [8]. In the structures of **107** and **108**, a hemiketal moiety was constructed in the 3-furanone ring but the stereochemistry at C-2 was unidentified so far (Figure 15). The chemical investigation of the same species mollusk *Siphonaria capensis* led to isolation of a 2-furanone-containing polypropionate capensifuranone (**109**) [9]. The all *S* configuration at the side chain was proposed by the same biosynthetic pathway with those of siphonarienfuranone and its *Z*-isomer, which were previously isolated from *S. lesson* and the absolute configuration was established by ozonolysis [61,62].

**Figure 16:**
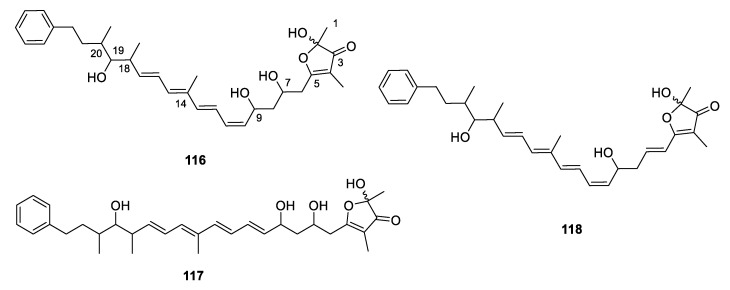
Chemical structures of **116**–**118**.

Six complex PPs, indoxamycins A–F (**110**–**115**) constructing a new 5/5/6 tricyclic system, were isolated from a marine-derived *Streptomyces* sp. (Figure 15). Compounds **110** and **115** exhibited significant growth inhibition against HT-29 tumor cell line with the IC_50_ values of 0.59 and 0.31 µM, respectively [63]. The myxobacterium *Hyalangium minutum* yielded three 3-furanone-containing derivatives hyafurones A1, A2 and B (**116**–**118**) (Figure 16) [23], of which the absolute configuration at C-2, C-7, C-9 C-18, C-19 and C-20 remained to be determined. The **116** could slowly convert to **117** when exposed to light or store in methanol. The antibacterial assays indicated that only **118** showed moderate activity against *Nocardia flava* (MIC = 8.3 µg/mL).

#### 2.2.4. Metabolites Containing both Pyran and Furan

**Figure 17:**
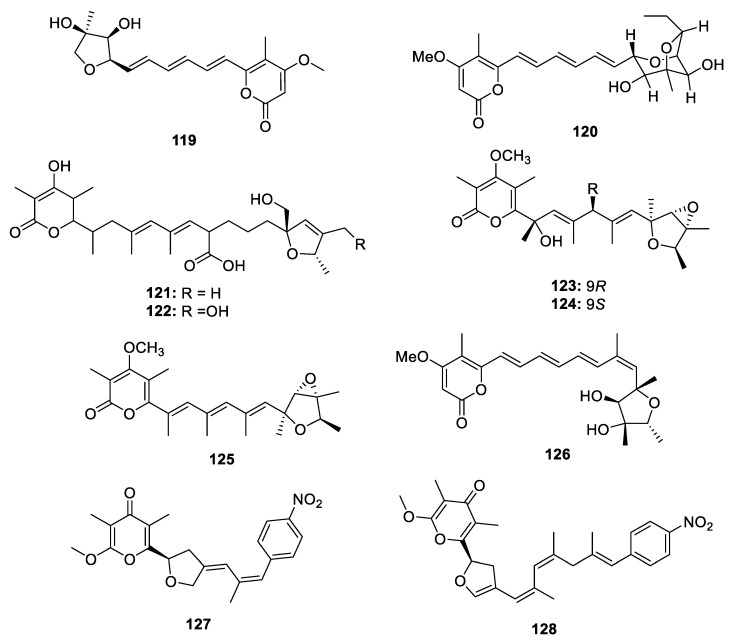
Chemical structures of **119**–**128**.

Ten PPs containing both pyran and furan rings were isolated from six species of fungi and two species of antinomycetes (Figure 17). Asteltoxin G (**119**) was isolated from a sponge-derived fungus, *Aspergillus ochraceopetaliformis* [64], and aurovertin E (**120**) was isolated from the mushroom *Albatrellus confluens* [65]. The heterologous expression of the *alp* gene cluster from *P. nodorum* not only produced the 2-pyrone PPs **54**–**56** but also gave two analogues, **121** and **122**, constructing a furan ring at the end of the side chain. **121** and **122** were detected to inhibit wheat germination significantly at 100 µg/mL [37]. The remaining four fungal-derived metabolites, penicyrones A and B (**123** and **124**), deoxyverrucosidin (**125**) and (+)-neocitreoviridin (**126**), were all isolated from *Penicillium* sp. [66,67,68] In the bioassays, **125** dose-dependently inhibited the expression of GRP78 promoter with an IC_50_ of 30 nM; meanwhile, **126** exhibited broad-spectrum antiviral effects against influenza A virus (IAV) (IC_50_ = 3.6 µM) and antibacterial activity against *Helicobacter pylori* (including drug-sensitive strain G27 and drug-resistant strain 159) with an MIC of 4 and 16 µg/mL, respectively. A new *p*-nitrophenyl-possessed polypropionate alloaureothin (**127**) with cytotoxicity against HT1080 was obtained from *Streptomyces* sp. MM23 [69]. Another *Streptomyces* sp. MK756-CF1 also yielded a similar analogue arabilin (**128**). Arabilin could competitively block the binding of androgen to the ligand-binding domain of AR in vitro; moreover, it could inhibit androgen-induced prostate-specific antigen mRNA expression in prostate cancer LNCaP cells [70].

#### 2.2.5. Other Heterocyclic Metabolites

**Figure 18:**
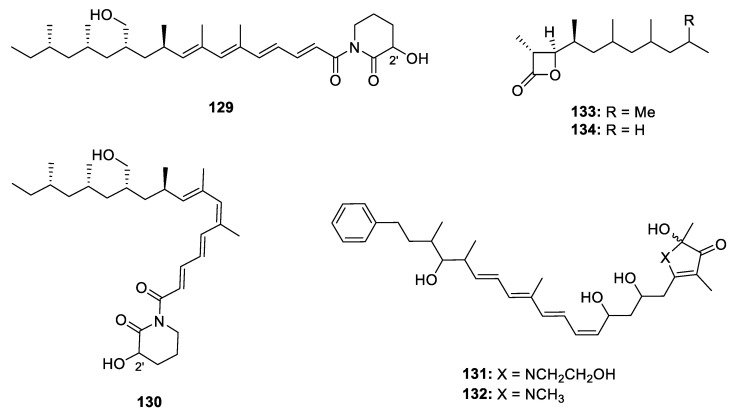
Chemical structures of **129**–**134**.

A few other unusual heterocyclic PPs were also discovered from nature (Figure 18). Two 3-hydroxypiperidin-2-one containing isomers at Δ^6^ (**129** and **130**) were produced by *A. fischeri*. This was the first example of polypropionate derivatives incorporating a 3-hydroxypiperidin-2-one via an imide linkage but the configuration of C-2’ remained unknown [10]. Like the other metabolites from this strain, **129** exhibited an *M*ptpB inhibitory effects with an IC_50_ of 11 µM. However, **130** did not show any activity at the concentration of 50 µM, suggesting that the geometry configuration changes will make a difference to the bioactivities. The myxobacterium *H. minutum* produced a series of heterocyclic PPs including two pyrrolidone-related derivatives hyapyrrolines A and B (**131** and **132**), which were the only members of propionates incorporating pyrrolidone to date [23]. Another two unusual four-membered lactone derivatives **133** and **134** were discovered from feeding male striped cucumber bettles *Acalymma vittatum*. The absolute configuration was assigned by modified Mosher’s method applied to the methanolysis products. An electrophysiological study indicated that **133** was possibly an aggregation pheromone for *A. vittatum* [71].

### 2.3. Macrocyclic Metabolites

The macrocyclic metabolites covered in this section are chosen to illustrate the structural features and biological activities because their biosynthetic pathway involves propionates. Twenty-six related derivatives were concluded belonging to two families: mangromicins family from an actinomycete and jaspamide family from mollusks.

**Figure 19:**
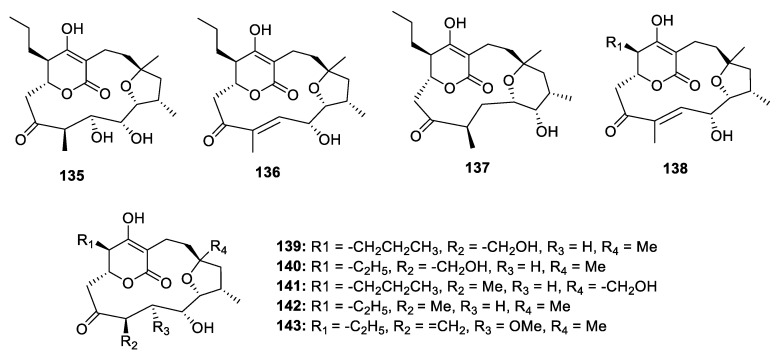
Chemical structures of **135**–**143**.

The mangromicin analogues possess a complicated cyclopentadecane skeleton and show antitrypanosomal and ROS scavenging activities (Figure 19). Takuji Nakashima and his co-workers discovered nine mangromicins A‒I (**135**–**143**) using a physical-chemical screening system in the actinomycete *Lechevalieria aerocolonigenes* K10-0216 [72,73,74]. Although an X-ray diffraction of **135** was carried out, the absolute configuration could not be determined by the unreliable Flack parameter—0.1 (3). Thus, only the relative configuration of mangromicins was assigned. **135** and **136** exhibited antitrypanosomal activities against *Trypanosoma brucei brucei* with the IC_50_ values of 2.4 and 43.4 µg/mL. Besides, except for **136**, **139** and **143**, all mangromicin analogs had more potent DPPH scavenging activity than α-tocopherol.

**Figure 20:**
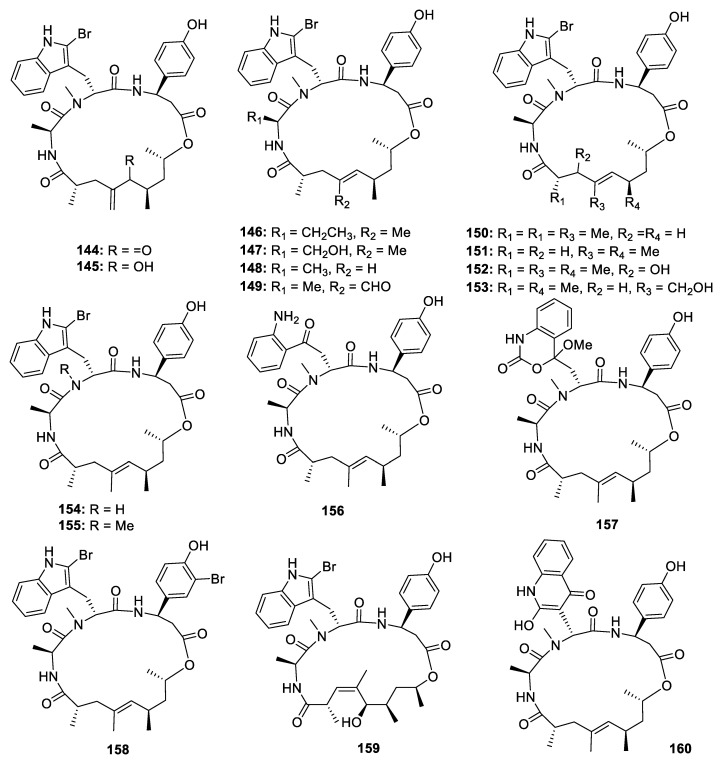
Chemical structures of **144**–**160**.

Jaspamide derivatives, with a propionate chain in the macro ring, are a group of cyclodepsipeptides driven from the NRPS-PKS hybrid biosynthetic pathway (Figure 20). In 1998 and 2008, Angela Zampella and his co-workers published four papers to describe the isolation and bioactivities of 14 jaspamide derivatives jaspamides B‒P (**144**–**157**) from the sponge *Jaspis splendans*. The stereochemistry was elucidated by comparing the NMR data with those of the metabolites containing the same fragments. All the jaspamides isolated from *J. splendans* exhibited significant cytotoxicities against different human cancer cell lines [75,76,77,78]. Chemical investigation of another Pacific marine sponge *Popestela candelabra* led to the isolation of another three unusual jaspamide analogues pipestelides A‒C (**158**–**160**), which contain a bromotyrosine [3-amino-3-(bromo-4-hydroxyphenyl)propanoic acid] unit, a polypropionate with a *Z* configuration at Δ^3^, and a 2-hydroxyquinolinone unit, respectively [79]. **158** exhibited strong inhibitory activities against the KB cell line with the IC_50_ value of 0.11 µM.

## 3. Summary

**Figure 21:**
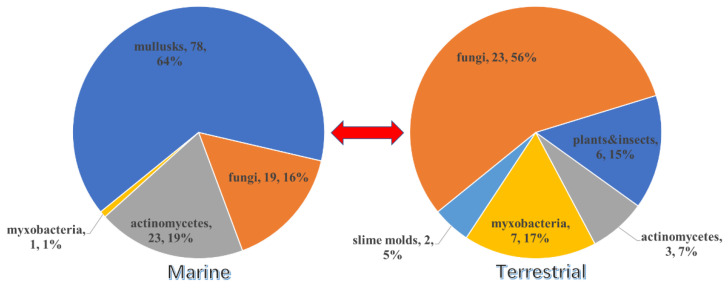
Sources of the 164 new PPs and the related fungal genes.

**Figure 22:**
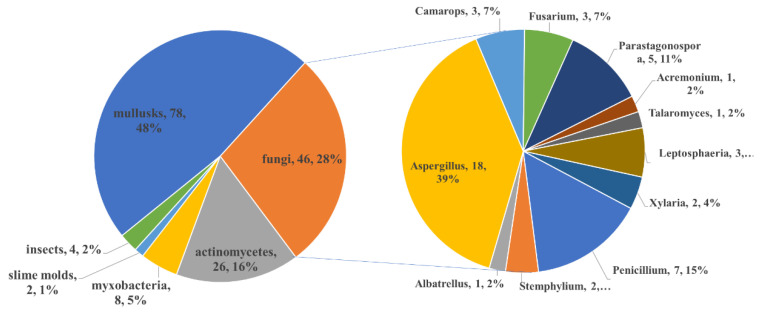
Comparison of the biodiversity between marine- and terrestrial-derived PPs.

Overall, except for the two PPs from unknown source-derived fungi, 121 of the 164 new PPs were isolated from the marine system, while 41 were produced by terrestrial organisms. It could be concluded that mollusks are the main producer among the marine organisms during the last two decades, which contributed 64% of the new marine-derived PPs (Figure 21) and nearly half (48%) of the total natural PPs (Figure 22). Fungi (containing marine-derived and terrestrial) provided the second largest number of the PPs (46 compounds accounting for 28% of total) and these fungi belonged to 11 genera including *Acremonium*, *Albatrellus*, *Aspergillus*, *Camarops*, *Fusarium*, *Penicillium*, *Leptosphaeria*, *Parastagonospora*, *Penicillium*, *Talaromyces* and *Xylaria*. The common *Aspergillus* and *Penicillium* are still the main producers of PPs. It is noticed that the fungi are the most important source in the terrestrial system (23 compounds accounting for 56%) compared to the marine system (19 compounds accounting for only 16%) (Figure 21). In the marine system, the actinomycetes are also the focused research producers, of which 23 PPs have been discovered.

**Figure 23:**
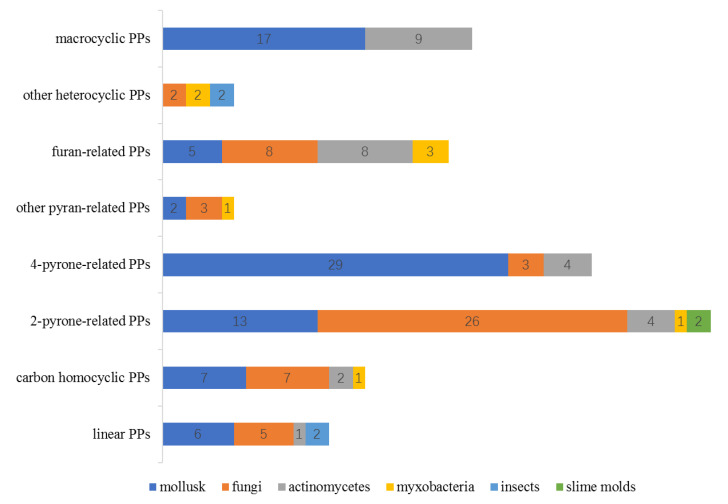
Relationship between the chemical features of PPs and their producers. The number in the bars represents the number of PPs from different resources.

The relationship between the chemical features of PPs and their producers was further analyzed. The compounds containing both pyran and furan-related moiety were counted in both the pyran- and furan-containing categories. As shown in Figure 23 and Table 1, mollusks performed the most abundant chemical diversity by producing all classes of PPs. The culturable fungi can also produce different classes of PPs except the macrocyclic PPs. It was noticed that mollusks contributed most of the 4-pyrone-containing PPs (81%) while fungi are the most important source of 2-pyrone-containing PPs (accounting for 58% of the total isolates). The speculated reason is that the mollusks synthesize PPs via a direct propionate polymerization [80,81], which is easy to form a 4-pyrone by 1,5-condensation. However, the PPs from fungi are generated by an acetate-methionine pathway [82] and the 2-pyrone was formed by the esterification at the terminal carboxyl group of the C_2_-generated polyketide chain. As far as fungal genera are concerned, the *Aspergillus* and *Penicillium* are the predominant fungal sources of PPs, accounting for 39% and 15% of the fungi-derived PPs, respectively.

**Figure 24:**
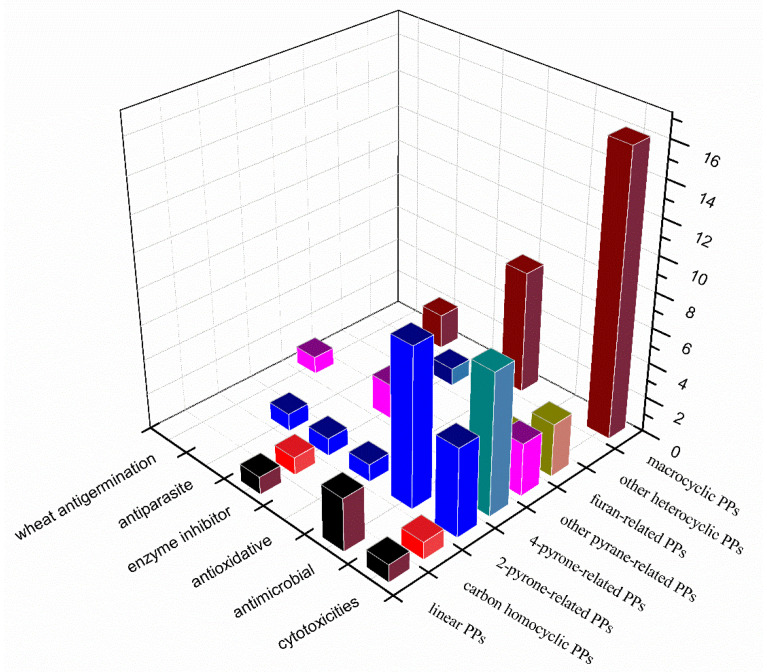
Relationship between the chemical classes of PPs and their bioactivities.

In the bioassays of the new isolated PPs, there are 69 metabolites (42% of total) exhibiting various activities, among which, cytotoxicity is the most significant pharmacological activity with 37 compounds exhibiting in vitro cytotoxicity against different tumor cell lines such as A549, HT1080, HeLa, etc. Then, 14 PPs were detected to show antimicrobial effects, including antibacterial activities for eight compounds, antifungal activities for five compounds and antiviral activity for one compound. The antioxidative activities (eight compounds) were mainly detected in macrocyclic metabolites of the mangromicin family. The other ten PPs exhibited activities including enzyme inhibition effects (six compounds), anti-parasite activities (three compounds) and wheat antigermination (one compound). A primary relationship between the chemical classes of the PPs and their bioactivities was summarized in Figure 24. It could be concluded that the macrocyclic metabolites were the most active among all classes of PPs since nearly all the isolated macrocyclic PPs were detected to show pharmacological activities. The pyran-related PPs showed the most extensive activities, of which the 4-pyrone-containing PPs were observed to exhibit cytotoxicity specifically.

## 4. Conclusions and Outlook

This review presents an overview of 164 PPs published in 67 research papers from 1999 to 2020, including their isolation, chemical features and bioactivities. The marine organisms (mainly the mollusks) are the dominant source of these PPs. It is worth noting that the culturable fungi and actinomycetes (not only from marine system but also from terrestrial resources) are becoming a more important source for the discovering of PPs. The modern molecular biological approaches including the genomic mining and heterologous expression will promote the rapid discovery of structural novel and biologically active PPs from fungi.

The assignment of the absolute configuration, especially the chiral centers in the side chain, remains a major obstacle for the structure identification of natural PPs. Nearly half of the natural PPs were reported without identifying the stereochemistry or with establishing the relative configuration only. In general, the configuration of the chiral centers at the cyclic core can be determined by NOE correlations and the quantum chemical calculation of ECD spectra, and the Mosher’s method is an effective approach to establish the hydroxyl-substituted chiral centers at the side chain. However, the assignment of methylated chiral centers at the side chain is the most difficult. Some methods such as chemical degradation, spectroscopic comparisons and semi-synthesis from the know precursors have been tried to solve the issue but are always limited by their instability or the small amount of the compounds obtained. The X-ray diffraction of a crystal with high-quality through Cu Kα can give a direct sight of the planar and absolute configuration; nevertheless, due to the flexibility of the side chain, the high-quality crystals of most PPs are still hard to obtain. In recent years, some advanced methods have been applied to solve the stereochemistry of the methylated side chain such as the C-H coupling constant, NMR data guided conformer searching, ^13^C NMR calculations, etc. Some interdisciplinary technologies, for example, the “crystal sponge” [83,84,85], will also provide some efficient solutions of the stereochemistry of PPs in the future. Moreover, inspired by their diverse complex structures and stereochemistry, an increasing total synthesis or semisynthesis of PPs has been carried out by chemists around the world [86].

Although nearly half of the natural PPs have been discovered to show potential pharmacological properties, few of them were selected to be lead compounds for further development of new drugs, which may be limited by the small amounts of compounds isolated from nature and the difficulty of obtaining the synthetic substitutes. Therefore, the multi-targeted screening and deeper biological mechanisms of PPs should be put on the agenda in the future.

The continuous studies toward the chemistry and biology of PPs will make an important contribution to the discovery of the lead compounds and the development of polypropionate-related drugs.

## Figures and Tables

**Table 1:** The number of PPs with different chemical features from different resources.

	Mollusk	Fungi	Actinomycetes	Myxobacter-ia	Insects	Slime Molds
linear PPs	6	5	1	-	2	-
carbon homocyclic PPs	7	7	2	1	-	-
2-pyrone-related PPs	13	26	4	1	-	2
4-pyrone-related PPs	29	3	4	-	-	-
other pyran-related PPs	2	3	-	1	-	-
furan-related PPs	5	8	8	3	-	-
other heterocyclic PPs	-	2	-	2	2	-
macrocyclic PPs	17	-	9	-	-	-

## References

[1] Müller W.E.G. (2006). Marine Molecular Biotechnology.

[2] Ireland C., Faulkner D.J. (1978). Tridachione, a propionate-derived metabolite of the opisthobranch mollusc *Tridachiella diomedea*. J. Am. Chem. Soc..

[3] Davies-Coleman M.T., Garson M.J. (1998). Marine polypropionates. Nat. Prod. Rep..

[4] Gunasekera S.P., Paul G.K., Longley R.E., Isbrucker R.A., Pomponi S.A. (2002). Five new discodermolide analogues from the marine sponge *Discodermia* species. J. Nat. Prod..

[5] Gunasekera S.P., Gunasekera M., Longley R.E., Schulte G.K. (1990). Discodermolide: A new bioactive polyhydroxylated lactone from the marine sponge *Discodermia dissolute*. J. Org. Chem..

[6] Nuzzo G., Cutignano A., Moles J., Avila C., Fontana A. (2016). Exiguapyrone and exiguaone, new polypropionates from the Mediterranean cephalaspidean mollusc *Haminoea exigua*. Tetrahedron Lett..

[7] Napolitano J.G., Souto M.L., Fernández J.J., Norte M. (2008). Micromelones A and B, noncontiguous polypropionates from *Micromelo undata*. J. Nat. Prod..

[8] Bromley C.L., Popplewell W.L., Pinchuck S.C., Hodgson A.N., Davies-Coleman M.T. (2012). Polypropionates from the South African marine mollusk *Siphonaria oculus*. J. Nat. Prod..

[9] Beukes D.R., Davies-Coleman M.T. (1999). Novel polypropionates from the South African marine mollusk *Siphonaria capensis*. Tetrahedron.

[10] Liu Z., Wang Q., Li S., Cui H., Sun Z., Chen D., Lu Y., Liu H., Zhang W. (2019). Polypropionate derivatives with *Mycobacterium tuberculosis* protein tyrosine phosphatase B inhibitory activities from the deep-sea-derived fungus *Aspergillus fischeri* FS452. J. Nat. Prod..

[11] Lin S., Wu Y.Z., Chen K.Y., Ye J., Yang X.W., Zhang W.D. (2018). Polyketides from the fungus *Penicillium decumbens*. J. Asian Nat. Prod. Res..

[12] Babadi Z.K., Sudarman E., Ebrahimipour G.H., Primahana G., Stadler M., Wink J. (2020). Structurally diverse metabolites from the rare actinobacterium *Saccharothrix xinjiangensis*. J. Antibiot..

[13] Jang Y.-W., Lee I.-K., Kim Y.-S., Lee S., Lee H.-J., Yu S., Yun B.-S. (2007). Xylarinic acids A and B, new antifungal polypropionates from the fruiting body of *Xylaria polymorpha*. J. Antibiot..

[14] Fletcher M.T., Chow S., Lambert L.K., Gallagher O.P., Cribb B.W., Allsopp P.G., Moore C.J., Kitching W. (2003). 4,6,8,10,16-Penta-and 4,6,8,10,16,18-hexamethyldocosanes from the cane beetle *Antitrogus parvulus*-cuticular hydrocarbons with unprecedented structure and stereochemistry. Org. Lett..

[15] Chow S., Fletcher M.T., Lambert L.K., Gallagher O.P., Moore C.J., Cribb B.W., Allsopp P.G., Kitching W. (2005). Novel cuticular hydrocarbons from the cane beetle *Antitrogus parvulus*-4,6,8,10,16-penta-and 4,6,8,10,16,18-hexamethyldocosaness-unprecedented *anti*-*anti*-*anti*-stereochemistry in the 4,6,8,10-methyltetrad. J. Org. Chem..

[16] Wu Q., Li S.-W., Xu H., Wang H., Hu P., Zhang H., Luo C., Chen K.-X., Nay B., Guo Y.-W. (2020). Complex polypropionates from a South China Sea photosynthetic mollusk: Isolation and biomimetic synthesis highlighting novel rearrangements. Angew. Chem. Int. Ed..

[17] Gavagnin M., Mollo E., Cimino G., Ortea J. (1996). A new γ-dihydropyrone-propionate from the Caribbean sacoglossan *Tridachia crispate*. Tetrahedron Lett..

[18] Neuhaus G.F., Adpressa D.A., Bruhn T., Loesgen S. (2019). Polyketides from marine-derived *Aspergillus porosus*: Challenges and opportunities for determining absolute configuration. J. Nat. Prod..

[19] Jouda J.B., Fopossi J.D., Kengne F.M., Djama Mbazoa C., Golz C., Strohmann C., Fogue S.K., Wandji J. (2017). Secondary metabolites from *Aspergillus japonicus* CAM231, an endophytic fungus associated with *Garcinia preussii*. Nat. Prod. Res..

[20] De Oliveira C.M., Silva G.H., Regasini L.O., Flausino O., Lopez S.N., Abissi B.M., Berlinck R.G., Sette L.D., Bonugli-Santos R.C., Rodrigues A. (2011). Xylarenones C-E from an endophytic fungus isolated from *Alibertia macrophylla*. J. Nat. Prod..

[21] Oh D.-C., Gontang E.A., Kauffman C.A., Jensen P.R., Fenical W. (2008). Salinipyrones and pacificanones, mixed-precursor polyketides from the marine actinomycete *Salinispora pacifica*. J. Nat. Prod..

[22] Sun Y., Tomura T., Sato J., Iizuka T., Fudou R., Ojika M. (2016). Isolation and biosynthetic analysis of haliamide, a new PKS-NRPS hybrid metabolite from the marine myxobacterium *Haliangium ochraceum*. Molecules.

[23] Okanya P.W., Mohr K.I., Gerth K., Kessler W., Jansen R., Stadler M., Muller R. (2014). Hyafurones, hyapyrrolines, and hyapyrones: Polyketides from *Hyalangium minutum*. J. Nat. Prod..

[24] Cutignano A., Blihoghe D., Fontana A., Villani G., d’Ippolito G., Cimino G. (2007). Fusaripyrones, novel polypropionates from the Mediterranean mollusc *Haminoea fusari*. Tetrahedron.

[25] Smith A.B., Lamarche M.L., Falcone-Hindley M. (2001). Solution structure of (+)-discodermolide. Org. Lett..

[26] Gunasekera S.P., Gunasekera M., Longley R.E., Schulte G.K. (1991). Discodermolide—A new bioactive polyhydroxylated lactone from the marine sponge *Discodermia-dissoluta*. J. Org. Chem..

[27] Gunasekera S.P., Cranick S., Longley R.E. (1989). Immunosuppressive compounds from a deep water marine sponge, *Agelas flabelliformis*. J. Nat. Prod..

[28] Longley R.E., Caddigan D., Harmody D., Gunasekera M., Gunasekera S.P. (1991). Discodermolide—A new, marine-derived immunosuppressive compound. I. In vitro studies. Transplantation.

[29] Longley R.E., Caddigan D., Harmody D., Gunasekera M., Gunasekera S.P., Gunasekera S.P. (1991). Discodermolide—A new, marine-derived immunosuppressive compound. II. In vitro studies. Transplantation.

[30] Longley R.E., Gunasekera S.P., Faherty D., McLane J., Dumont F. (1993). Immunosuppression by discodermolide. Ann. N. Y. Acad. Sci..

[31] Ding L., Ren L., Li S., Song J., Han Z., He S., Xu S. (2019). Production of new antibacterial 4-hydroxy-alpha-pyrones by a marine fungus *Aspergillus niger* cultivated in solid medium. Mar. Drugs.

[32] Li T.X., Meng D.D., Wang Y., An J.L., Bai J.F., Jia X.W., Xu C.P. (2020). Antioxidant coumarin and pyrone derivatives from the insect-associated fungus *Aspergillus versicolor*. Nat. Prod. Res..

[33] Yamazaki H., Takahashi K., Iwakura N., Abe T., Akaishi M., Chiba S., Namikoshi M., Uchida R. (2018). A new protein tyrosine phosphatase 1B inhibitory alpha-pyrone-type polyketide from Okinawan plant-associated *Aspergillus* sp. TMPU1623. J. Antibiot..

[34] Gao H., Li G., Peng X.P., Lou H.X. (2020). Fupyrones A and B, two new alpha-pyrones from an endophytic fungus, *Fusarium* sp. F20. Nat. Prod. Res..

[35] Pedras M.S., Chumala P.B. (2005). Phomapyrones from blackleg causing phytopathogenic fungi: Isolation, structure determination, biosyntheses and biological activity. Phytochemistry.

[36] Asai T., Luo D., Yamashita K., Oshima Y. (2013). Structures and biomimetic synthesis of novel α-pyrone polyketides of an endophytic *Penicillium* sp. in *Catharanthus roseus*. Org. Lett..

[37] Li H., Hu J., Wei H., Solomon P.S., Vuong D., Lacey E., Stubbs K.A., Piggott A.M., Chooi Y.-H. (2018). Chemical ecogenomics-guided discovery of phytotoxic α-pyrones from the fungal wheat pathogen *Parastagonospora nodorum*. Org. Lett..

[38] Zhou X.M., Zheng C.J., Song X.P., Han C.R., Chen W.H., Chen G.Y. (2014). Antibacterial alpha-pyrone derivatives from a mangrove-derived fungus *Stemphylium* sp. 33231 from the South China Sea. J. Antibiot..

[39] Tomikawa T., Shin-ya K., Furihata K., Kinoshita T., Miyajima A., Seto H., Hayakawa Y. (2000). Rasfonin, a new apoptosis inducer in ras-dependent cells from *Talaromyces* sp.. J. Antibiot..

[40] Sato S., Iwata F., Yamada S., Kawahara H. (2011). 3,6,7-Tri-epi-invictolide, a diastereomer of queen recognition pheromone, and its analog from a marine-derived actinomycete. J. Antibiot..

[41] Řezanka T., Dvořáková R. (2003). Polypropionate lactones of deoxysugars glycosides from slime mold *Lycogala epidendrum*. Phytochemistry.

[42] Cueto M., D’Croz L., Maté J.L., San-Martín A., Darias J. (2005). Elysiapyrones from *Elysia diomedea*. Do such metabolites evidence an enzymatically assisted electrocyclization cascade for the biosynthesis of their bicyclo[4.2.0]octane core?. Org. Lett..

[43] Manzo E., Ciavatta M.L., Gavagnin M., Mollo E., Wahidulla S., Cimino G. (2005). New γ-pyrone propionates from the Indian Ocean sacoglossan *Placobranchus ocellatus*. Tetrahedron Lett..

[44] Miller A.K., Trauner D. (2005). Mining the tetraene manifold: Total synthesis of complex pyrones from *Placobranchus ocellatus*. Angew. Chem. Int. Ed..

[45] Carbone M., Muniain C., Castelluccio F., Iannicelli O., Gavagnin M. (2013). First chemical study of the sacoglossan *Elysia patagonica*: Isolation of a γ-pyrone propionate hydroperoxide. Biochem. Syste. Ecol..

[46] Carbone M., Ciavatta M.L., Wang J.R., Cirillo I., Mathieu V., Kiss R., Mollo E., Guo Y.W., Gavagnin M. (2013). Extending the record of bis-gamma-pyrone polypropionates from marine pulmonate mollusks. J. Nat. Prod..

[47] Zhou Z.F., Li X.L., Yao L.G., Li J., Gavagnin M., Guo Y.W. (2018). Marine bis-gamma-pyrone polypropionates of onchidione family and their effects on the XBP1 gene expression. Bioorg. Med. Chem. Lett..

[48] Carbone M., Gavagnin M., Mattia C.A., Lotti C., Castelluccio F., Pagano B., Mollo E., Guo Y.-W., Cimino G. (2009). Structure of onchidione, a bis-γ-pyrone polypropionate from a marine pulmonate mollusk. Tetrahedron.

[49] Wang J.-R., Carbone M., Gavagnin M., Mándi A., Antus S., Yao L.-G., Cimino G., Kurtán T., Guo Y.-W. (2012). Assignment of absolute configuration of bis-γ-pyrone polypropionates from marine pulmonate molluscs. Eur. J. Org. Chem..

[50] Chen D.L., Zheng W., Feng J., Ma G.X., Liu Y.Y., Xu X.D. (2019). A new bis-gamma-pyrone polypropionate from a marine pulmonate mollusc *Onchidium struma*. J. Asian. Nat. Prod. Res..

[51] Li S.W., Cuis W.X., Huan X.J., Gavagnin M., Mollo E., Miao Z.H., Yao L.G., Li X.W., Guo Y.W. (2020). A new bis-gamma-pyrone polypropionate of onchidiol family from marine pulmonate mollusk *Onchidium* sp.. Nat. Prod. Res..

[52] Fu X., Hong E.P., Schmitz F.J. (2000). New polypropionate pyrones from the Philippine sacoglossan mollusk *Placobranchus ocellatus*. Tetrahedron.

[53] Cutignano A., Fontana A., Rennzulli L., Cimino G. (2003). Placidenes C-F, novel α-pyrone propionates from the Mediterranean Sacoglossan *Placida dendritica*. J. Nat. Prod..

[54] Brecknell D.J., Collett L.A., Davies-Coleman M.T., Garson M.J., Jones D.D. (2000). New non-contiguous polypropionates from marine molluscs: A comment on their natural products status. Tetrahedron.

[55] Esposito G., Teta R., Della Sala G., Pawlik J.R., Mangoni A., Costantino V. (2018). Isolation of smenopyrone, a bis-gamma-pyrone polypropionate from the Caribbean sponge *Smenospongia aurea*. Mar. Drugs.

[56] Peng X.P., Li G., Ji L.X., Li Y.X., Lou H.X. (2020). Acrepyrone A, a new gamma-pyrone derivative from an endophytic fungus, *Acremonium citrinum* SS-g13. Nat. Prod. Res..

[57] Choi H.G., Song J.H., Park M., Kim S., Kim C.E., Kang K.S., Shim S.H. (2020). Neuroprotective gamma-pyrones from *Fusarium Solani* JS-0169: Cell-based identification of active compounds and an informatics approach to predict the mechanism of action. Biomolecules.

[58] Hayakawa Y., Saito J., Izawa M., Shin-ya K. (2014). Actinopyrone D, a new downregulator of the molecular chaperone GRP78 from *Streptomyces* sp.. J. Antibiot..

[59] Liu S.H., Xu M.D., Zhang H., Qi H., Zhang J., Liu C.X., Wang J.D., Xiang W.S., Wang X.J. (2016). New cytotoxic spectinabilin derivative from ant-associated *Streptomyces* sp. 1H-GS5. J. Antibiot..

[60] Jimenez-Romero C., Gonzalez K., Rodriguez A.D. (2012). Dolabriferols B and C, non-contiguous polypropionate esters from the tropical sea hare *Dolabrifera dolabrifera*. Tetrahedron Lett..

[61] Rovirosa J., Quezada E. (1991). San-Martin, new polypropionates of *Siphonaria-lessoni* from Chilean coasts. Bol. Soc. Chil. Quim..

[62] Capon R.J., Faulkner D.J. (1984). Metabolites of the pulmonate *Siphonaria lessoni*. J. Org. Chem..

[63] Sato S., Iwata F., Mukai T., Yamada S., Takeo J., Abe A., Kawahara H. (2009). Indoxamycins A-F. cytotoxic tricycklic polypropionates from a marine-derived actinomycete. J. Org. Chem..

[64] Liu J.T., Wu W., Cao M.J., Yang F., Lin H.W. (2018). Trienic alpha-pyrone and ochratoxin derivatives from a sponge-derived fungus *Aspergillus ochraceopetaliformis*. Nat. Prod. Res..

[65] Wang F., Luo D.-Q., Liu J.-L. (2005). Aurovertin E, a new polyene pyrone from the basidiomycete *Albatrellus confluens*. J. Antibiot..

[66] Bu Y.Y., Yamazaki H., Takahashi O., Kirikoshi R., Ukai K., Namikoshi M. (2016). Penicyrones A and B, an epimeric pair of alpha-pyrone-type polyketides produced by the marine-derived *Penicillium* sp.. J. Antibiot..

[67] Choo S.-J., Park H.-R., Ryoo J.-J., Kim J.-P., Yun B.-S., Kim C.-J., Shim-ya K., Yoo I.-D. (2005). Deoxyverrucosidin, a novel GRP78/BiP down-regulator, produced by *Penicillium* sp.. J. Antibiot..

[68] Li J., Wang Y., Hao X., Li S., Jia J., Guan Y., Peng Z., Bi H., Xiao C., Cen S. (2019). Broad-spectrum antiviral natural products from the marine-derived *Penicillium* sp. IMB17-046. Molecules.

[69] Ueda J., Hashimoto J., Nagai A., Nakashima T., Komaki H., Anzai K., Harayama S., Doi T., Takahashi T., Nagasawa K. (2007). New aureothin derivative, alloaureothin, from *Streptomyces* sp. MM23. J. Antibiot..

[70] Kawamura T., Fujimaki T., Hamanaka N., Torii K., Kobayashi H., Takahashi Y., Igarashi M., Kinoshita N., Nishimura Y., Tashiro E. (2010). Isolation and structure elucidation of a novel androgen antagonist, arabilin, produced by *Streptomyces* sp. MK756-CF1. J. Antibiot..

[71] Morris B.D., Smyth R.R., Foster S.P., Hoffmann M.P., Roelofs W.L., Franke S., Francke W. (2005). Vittatalactone, a β-lactone from the striped cucumber beetle, *Acalymma vittatum*. J. Nat. Prod..

[72] Nakashima T., Kamiya Y., Iwatsuki M., Takahashi Y., Omura S. (2014). Mangromicins, six new anti-oxidative agents isolated from a culture broth of the actinomycete, *Lechevalieria aerocolonigenes* K10-0216. J. Antibiot..

[73] Nakashima T., Iwatsuki M., Ochiai J., Kamiya Y., Nagai K., Matsumoto A., Ishiyama A., Otoguro K., Shiomi K., Takahashi Y. (2014). Mangromicins A and B: Structure and antitrypanosomal activity of two new cyclopentadecane compounds from *Lechevalieria aerocolonigenes* K10-0216. J. Antibiot..

[74] Nakashima T., Kamiya Y., Iwatsuki M., Sato N., Takahashi Y., Omura S. (2015). Mangromicin C, a new analog of mangromicin. J. Antibiot..

[75] Zampella A., Giannini C., Debitus C., Roussakis C., D’Auria V. (1999). New jaspamide derivatives from the marine sponge *Jaspis splendans* collected in Vanuatu. J. Nat. Prod..

[76] Gala F., D’Auria M.V., De Marino S., Zollo F., Smith C.D., Copper J.E., Zampella A. (2007). New jaspamide derivatives with antimicrofilament activity from the sponge *Jaspis splendans*. Tetrahedron.

[77] Gala F., D’Auria M.V., De Marino S., Sepe V., Zollo F., Smith C.D., Copper J.E., Zampella A. (2008). Jaspamides H–L, new actin-targeting depsipeptides from the sponge *Jaspis splendans*. Tetrahedron.

[78] Gala F., D’Auria M.V., De Marino S., Sepe V., Zollo F., Smith C.D., Keller S.N., Zampella A. (2009). Jaspamides M–P: New tryptophan modified jaspamide derivatives from the sponge *Jaspis splendans*. Tetrahedron.

[79] Sorres J., Martin M.T., Petek S., Levaique H., Cresteil T., Ramos S., Thoison O., Debitus C., Al-Mourabit A. (2012). Pipestelides A-C: Cyclodepsipeptides from the Pacific marine sponge *Pipestela candelabra*. J. Nat. Prod..

[80] Cimino G., Sodano G. (1993). Marine Natural Products−Diversity and Biosynthesis.

[81] Fontana A., Manzo E., Ciavatta M.L., Cutignano A., Gavagnin M., Cimino G., Fattorusso E., Gerwick W.H., Taglialatela-Scafati (2012). Biosynthetic studies through feeling experiments in marine organisms. Handbook of Marine Natural Products.

[82] Pfeifer B.A., Khosla C. (2001). Biosynthesis of polyketides in heterologous hosts. Micobiol. Mol. Biol. Rev..

[83] Inokuma Y., Yoshioka S., Ariyoshim J., Arai T., Hitora Y., Takada K., Matsunaga S., Rissanen K., Fujita M. (2013). X-ray analysis on the nanogram to microgram scale using porous complexes. Nature.

[84] Inokuma Y., Yoshioka S., Ariyoshi J., Arai T., Fujita M. (2014). Preparation and guest-uptake protocol for a porous complex useful for ‘crystal-free’ crystallography. Nat. Protoc..

[85] Yoshioka S., Inokuma Y., Hoshino M., Sato T., Fujita M. (2015). Absolute structure determination of compounds with axial and planar chirality using the crystalline sponge method. Chem. Sci..

[86] Turks M., Laclef S., Vogel P. (2013). Construction of polypropionate fragments in natural product synthesis. Stereoselective Synthesis of Drugs and Natural Products.

